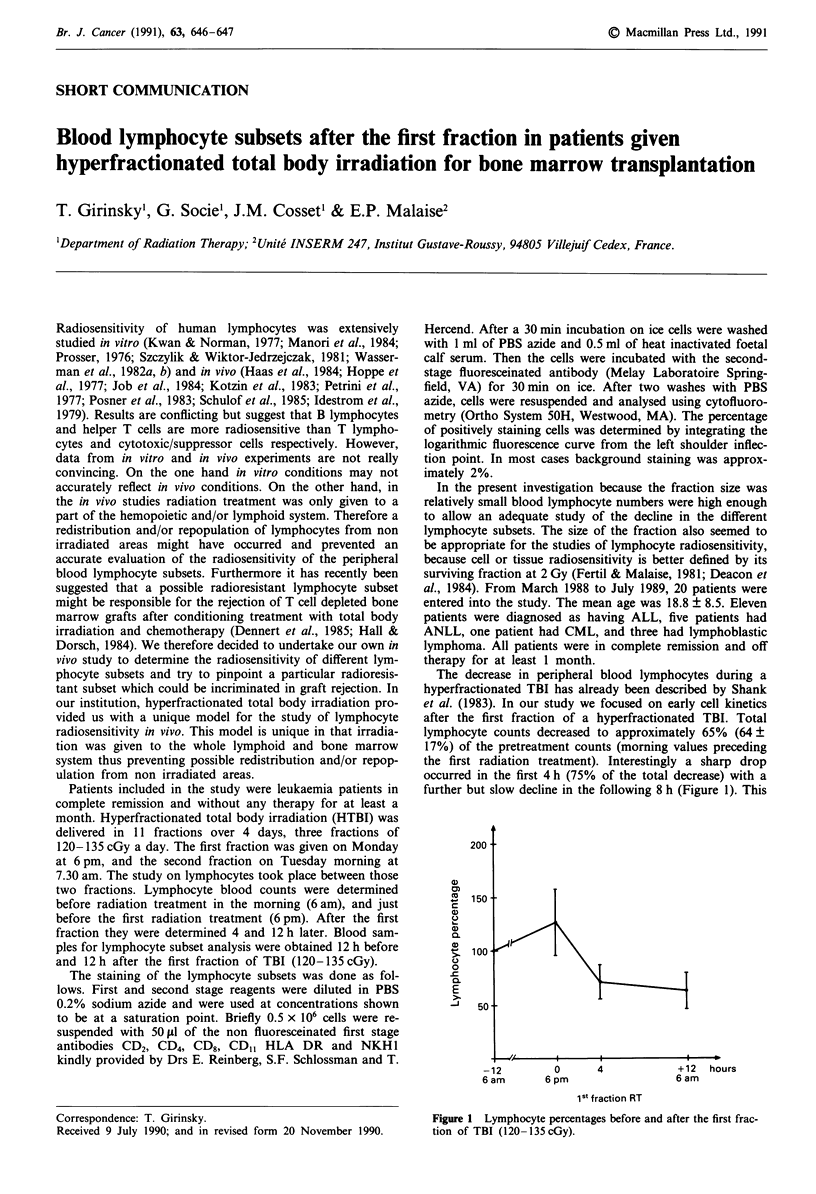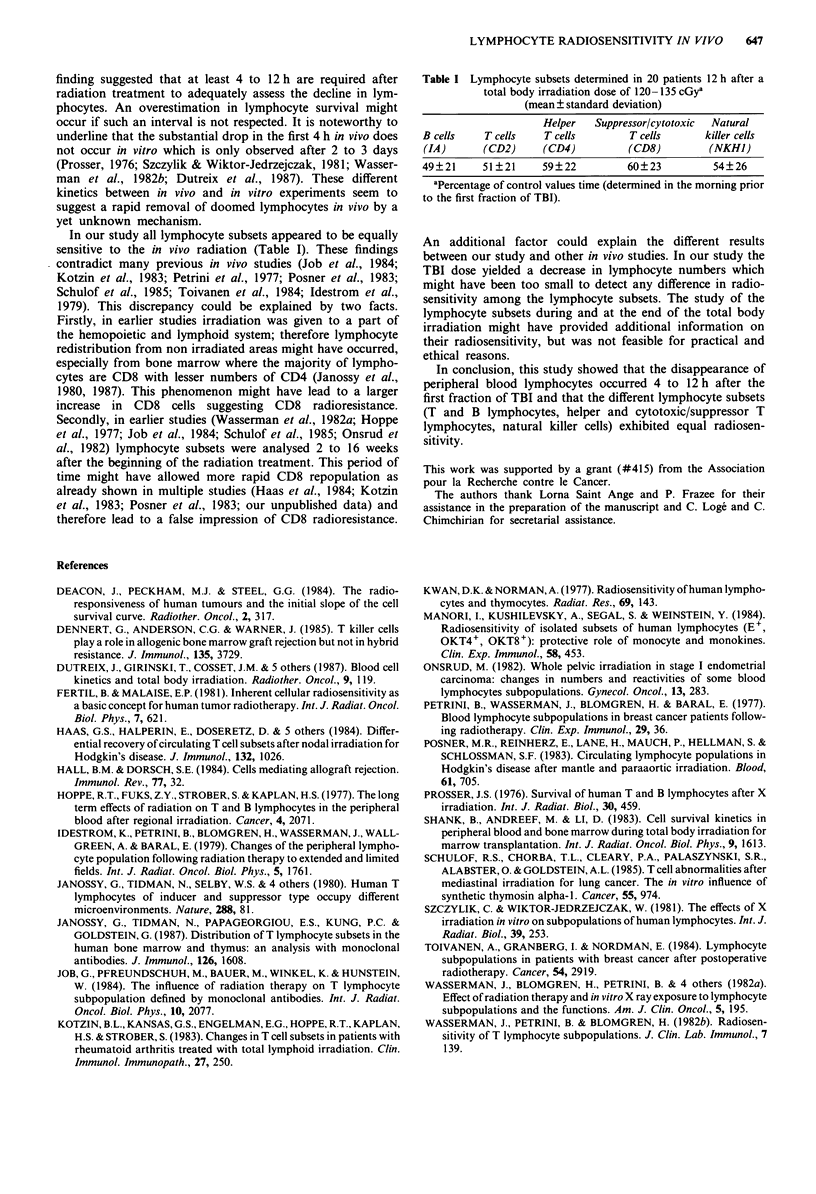# Blood lymphocyte subsets after the first fraction in patients given hyperfractionated total body irradiation for bone marrow transplantation.

**DOI:** 10.1038/bjc.1991.148

**Published:** 1991-04

**Authors:** T. Girinsky, G. Socie, J. M. Cosset, E. P. Malaise

**Affiliations:** Department of Radiation Therapy, Institut Gustave-Roussy, Villejuif, France.


Br. J. Cancer (1991), 63, 646–647 © Macmillan Press Ltd., 1991

SHORT COMMUNICATION

### Blood lymphocyte subsets after the first fraction in patients given hyperfractionated total body irradiation for bone marrow transplantation

T. Girinsky[1], G. Socie[1], J.M. Cosset[1] & E.P. Malaise[2]

[1]Department of Radiation Therapy; [2]Unité INSERM 247, Institut Gustave-Roussy, 94805 Villejuif Cedex, France.

Radiosensitivity of human lymphocytes was extensively studied *in vitro* (Kwan & Norman, 1977; Manori *et al.*, 1984; Prosser, 1976; Szczylik & Wiktor-Jedrzejczak, 1981; Wasserman *et al.*, 1982*a*, *b*) and *in vivo* (Haas *et al.*, 1984; Hoppe *et al.*, 1977; Job *et al.*, 1984; Kotzin *et al.*, 1983; Petrini *et al.*, 1977; Posner *et al.*, 1983; Schulof *et al.*, 1985; Idestrom *et al.*, 1979). Results are conflicting but suggest that B lymphocytes and helper T cells are more radiosensitive than T lymphocytes and cytotoxic/suppressor cells respectively. However, data from *in vitro* and *in vivo* experiments are not really convincing. On the one hand *in vitro* conditions may not accurately reflect *in vivo* conditions. On the other hand, in the *in vivo* studies radiation treatment was only given to a part of the hemopoietic and/or lymphoid system. Therefore a redistribution and/or repopulation of lymphocytes from non irradiated areas might have occurred and prevented an accurate evaluation of the radiosensitivity of the peripheral blood lymphocyte subsets. Furthermore it has recently been suggested that a possible radioresistant lymphocyte subset might be responsible for the rejection of T cell depleted bone marrow grafts after conditioning treatment with total body irradiation and chemotherapy (Dennert *et al.*, 1985; Hall & Dorsch, 1984). We therefore decided to undertake our own *in vivo* study to determine the radiosensitivity of different lymphocyte subsets and try to pinpoint a particular radioresistant subset which could be incriminated in graft rejection. In our institution, hyperfractionated total body irradiation provided us with a unique model for the study of lymphocyte radiosensitivity *in vivo*. This model is unique in that irradiation was given to the whole lymphoid and bone marrow system thus preventing possible redistribution and/or repopulation from non irradiated areas.

Patients included in the study were leukaemia patients in complete remission and without any therapy for at least a month. Hyperfractionated total body irradiation (HTBI) was delivered in 11 fractions over 4 days, three fractions of 120–135 cGy a day. The first fraction was given on Monday at 6 pm, and the second fraction on Tuesday morning at 7.30 am. The study on lymphocytes took place between those two fractions. Lymphocyte blood counts were determined before radiation treatment in the morning (6 am), and just before the first radiation treatment (6 pm). After the first fraction they were determined 4 and 12 h later. Blood samples for lymphocyte subset analysis were obtained 12 h before and 12 h after the first fraction of TBI (120–135 cGy).

The staining of the lymphocyte subsets was done as follows. First and second stage reagents were diluted in PBS 0.2% sodium azide and were used at concentrations shown to be at a saturation point. Briefly $0.5 \times 10^6$ cells were resuspended with 50 µl of the non fluoresceinated first stage antibodies $CD_2$, $CD_4$, $CD_8$, $CD_{11}$ HLA DR and NKH1 kindly provided by Drs E. Reinberg, S.F. Schlossman and T. Hercend. After a 30 min incubation on ice cells were washed with 1 ml of PBS azide and 0.5 ml of heat inactivated foetal calf serum. Then the cells were incubated with the second-stage fluoresceinated antibody (Melay Laboratoire Springfield, VA) for 30 min on ice. After two washes with PBS azide, cells were resuspended and analysed using cytofluorometry (Ortho System 50H, Westwood, MA). The percentage of positively staining cells was determined by integrating the logarithmic fluorescence curve from the left shoulder inflection point. In most cases background staining was approximately 2%.

In the present investigation because the fraction size was relatively small blood lymphocyte numbers were high enough to allow an adequate study of the decline in the different lymphocyte subsets. The size of the fraction also seemed to be appropriate for the studies of lymphocyte radiosensitivity, because cell or tissue radiosensitivity is better defined by its surviving fraction at 2 Gy (Fertil & Malaise, 1981; Deacon *et al.*, 1984). From March 1988 to July 1989, 20 patients were entered into the study. The mean age was 18.8 ± 8.5. Eleven patients were diagnosed as having ALL, five patients had ANLL, one patient had CML, and three had lymphoblastic lymphoma. All patients were in complete remission and off therapy for at least 1 month.

The decrease in peripheral blood lymphocytes during a hyperfractionated TBI has already been described by Shank *et al.* (1983). In our study we focused on early cell kinetics after the first fraction of a hyperfractionated TBI. Total lymphocyte counts decreased to approximately 65% (64 ± 17%) of the pretreatment counts (morning values preceding the first radiation treatment). Interestingly a sharp drop occurred in the first 4 h (75% of the total decrease) with a further but slow decline in the following 8 h (Figure 1). This

**Figure 1** Lymphocyte percentages before and after the first fraction of TBI (120–135 cGy).

Correspondence: T. Girinsky.
Received 9 July 1990; and in revised form 20 November 1990.

finding suggested that at least 4 to 12 h are required after radiation treatment to adequately assess the decline in lymphocytes. An overestimation in lymphocyte survival might occur if such an interval is not respected. It is noteworthy to underline that the substantial drop in the first 4 h *in vivo* does not occur *in vitro* which is only observed after 2 to 3 days (Prosser, 1976; Szczylik & Wiktor-Jedrzejczak, 1981; Wasserman *et al.*, 1982*b*; Dutreix *et al.*, 1987). These different kinetics between *in vivo* and *in vitro* experiments seem to suggest a rapid removal of doomed lymphocytes *in vivo* by a yet unknown mechanism.

In our study all lymphocyte subsets appeared to be equally sensitive to the *in vivo* radiation (Table I). These findings contradict many previous *in vivo* studies (Job *et al.*, 1984; Kotzin *et al.*, 1983; Petrini *et al.*, 1977; Posner *et al.*, 1983; Schulof *et al.*, 1985; Toivanen *et al.*, 1984; Idestrom *et al.*, 1979). This discrepancy could be explained by two facts. Firstly, in earlier studies irradiation was given to a part of the hemopoietic and lymphoid system; therefore lymphocyte redistribution from non irradiated areas might have occurred, especially from bone marrow where the majority of lymphocytes are CD8 with lesser numbers of CD4 (Janossy *et al.*, 1980, 1987). This phenomenon might have lead to a larger increase in CD8 cells suggesting CD8 radioresistance. Secondly, in earlier studies (Wasserman *et al.*, 1982*a*; Hoppe *et al.*, 1977; Job *et al.*, 1984; Schulof *et al.*, 1985; Onsrud *et al.*, 1982) lymphocyte subsets were analysed 2 to 16 weeks after the beginning of the radiation treatment. This period of time might have allowed more rapid CD8 repopulation as already shown in multiple studies (Haas *et al.*, 1984; Kotzin *et al.*, 1983; Posner *et al.*, 1983; our unpublished data) and therefore lead to a false impression of CD8 radioresistance.

**Table I** Lymphocyte subsets determined in 20 patients 12 h after a total body irradiation dose of 120–135 cGy[a] (mean ± standard deviation)

| *B cells (IA)* | *T cells (CD2)* | *Helper T cells (CD4)* | *Suppressor/cytotoxic T cells (CD8)* | *Natural killer cells (NKH1)* |
|---|---|---|---|---|
| 49±21 | 51±21 | 59±22 | 60±23 | 54±26 |

[a]Percentage of control values time (determined in the morning prior to the first fraction of TBI).

An additional factor could explain the different results between our study and other *in vivo* studies. In our study the TBI dose yielded a decrease in lymphocyte numbers which might have been too small to detect any difference in radiosensitivity among the lymphocyte subsets. The study of the lymphocyte subsets during and at the end of the total body irradiation might have provided additional information on their radiosensitivity, but was not feasible for practical and ethical reasons.

In conclusion, this study showed that the disappearance of peripheral blood lymphocytes occurred 4 to 12 h after the first fraction of TBI and that the different lymphocyte subsets (T and B lymphocytes, helper and cytotoxic/suppressor T lymphocytes, natural killer cells) exhibited equal radiosensitivity.

This work was supported by a grant (#415) from the Association pour la Recherche contre le Cancer.

The authors thank Lorna Saint Ange and P. Frazee for their assistance in the preparation of the manuscript and C. Logé and C. Chimchirian for secretarial assistance.

### References

DEACON, J., PECKHAM, M.J. & STEEL, G.G. (1984). The radioresponsiveness of human tumours and the initial slope of the cell survival curve. *Radiother. Oncol.*, **2**, 317.

DENNERT, G., ANDERSON, C.G. & WARNER, J. (1985). T killer cells play a role in allogenic bone marrow graft rejection but not in hybrid resistance. *J. Immunol.*, **135**, 3729.

DUTREIX, J., GIRINSKI, T., COSSET, J.M. & 5 others (1987). Blood cell kinetics and total body irradiation. *Radiother. Oncol.*, **9**, 119.

FERTIL, B. & MALAISE, E.P. (1981). Inherent cellular radiosensitivity as a basic concept for human tumor radiotherapy. *Int. J. Radiat. Oncol. Biol. Phys.*, **7**, 621.

HAAS, G.S., HALPERIN, E., DOSERETZ, D. & 5 others (1984). Differential recovery of circulating T cell subsets after nodal irradiation for Hodgkin's disease. *J. Immunol.*, **132**, 1026.

HALL, B.M. & DORSCH, S.E. (1984). Cells mediating allograft rejection. *Immunol. Rev.*, **77**, 32.

HOPPE, R.T., FUKS, Z.Y., STROBER, S. & KAPLAN, H.S. (1977). The long term effects of radiation on T and B lymphocytes in the peripheral blood after regional irradiation. *Cancer*, **4**, 2071.

IDESTROM, K., PETRINI, B., BLOMGREN, H., WASSERMAN, J., WALLGREEN, A. & BARAL, E. (1979). Changes of the peripheral lymphocyte population following radiation therapy to extended and limited fields. *Int. J. Radiat. Oncol. Biol. Phys.*, **5**, 1761.

JANOSSY, G., TIDMAN, N., SELBY, W.S. & 4 others (1980). Human T lymphocytes of inducer and suppressor type occupy different microenvironments. *Nature*, **288**, 81.

JANOSSY, G., TIDMAN, N., PAPAGEORGIOU, E.S., KUNG, P.C. & GOLDSTEIN, G. (1987). Distribution of T lymphocyte subsets in the human bone marrow and thymus: an analysis with monoclonal antibodies. *J. Immunol.*, **126**, 1608.

JOB, G., PFREUNDSCHUH, M., BAUER, M., WINKEL, K. & HUNSTEIN, W. (1984). The influence of radiation therapy on T lymphocyte subpopulation defined by monoclonal antibodies. *Int. J. Radiat. Oncol. Biol. Phys.*, **10**, 2077.

KOTZIN, B.L., KANSAS, G.S., ENGELMAN, E.G., HOPPE, R.T., KAPLAN, H.S. & STROBER, S. (1983). Changes in T cell subsets in patients with rheumatoid arthritis treated with total lymphoid irradiation. *Clin. Immunol. Immunopath.*, **27**, 250.

KWAN, D.K. & NORMAN, A. (1977). Radiosensitivity of human lymphocytes and thymocytes. *Radiat. Res.*, **69**, 143.

MANORI, I., KUSHILEVSKY, A., SEGAL, S. & WEINSTEIN, Y. (1984). Radiosensitivity of isolated subsets of human lymphocytes ($E^+$, $OKT4^+$, $OKT8^+$): protective role of monocyte and monokines. *Clin. Exp. Immunol.*, **58**, 453.

ONSRUD, M. (1982). Whole pelvic irradiation in stage I endometrial carcinoma: changes in numbers and reactivities of some blood lymphocytes subpopulations. *Gynecol. Oncol.*, **13**, 283.

PETRINI, B., WASSERMAN, J., BLOMGREN, H. & BARAL, E. (1977). Blood lymphocyte subpopulations in breast cancer patients following radiotherapy. *Clin. Exp. Immunol.*, **29**, 36.

POSNER, M.R., REINHERZ, E., LANE, H., MAUCH, P., HELLMAN, S. & SCHLOSSMAN, S.F. (1983). Circulating lymphocyte populations in Hodgkin's disease after mantle and paraaortic irradiation. *Blood*, **61**, 705.

PROSSER, J.S. (1976). Survival of human T and B lymphocytes after X irradiation. *Int. J. Radiat. Biol.*, **30**, 459.

SHANK, B., ANDREEF, M. & LI, D. (1983). Cell survival kinetics in peripheral blood and bone marrow during total body irradiation for marrow transplantation. *Int. J. Radiat. Oncol. Biol. Phys.*, **9**, 1613.

SCHULOF, R.S., CHORBA, T.L., CLEARY, P.A., PALASZYNSKI, S.R., ALABSTER, O. & GOLDSTEIN, A.L. (1985). T cell abnormalities after mediastinal irradiation for lung cancer. The *in vitro* influence of synthetic thymosin alpha-1. *Cancer*, **55**, 974.

SZCZYLIK, C. & WIKTOR-JEDRZEJCZAK, W. (1981). The effects of X irradiation *in vitro* on subpopulations of human lymphocytes. *Int. J. Radiat. Biol.*, **39**, 253.

TOIVANEN, A., GRANBERG, I. & NORDMAN, E. (1984). Lymphocyte subpopulations in patients with breast cancer after postoperative radiotherapy. *Cancer*, **54**, 2919.

WASSERMAN, J., BLOMGREN, H., PETRINI, B. & 4 others (1982*a*). Effect of radiation therapy and *in vitro* X ray exposure to lymphocyte subpopulations and the functions. *Am. J. Clin. Oncol.*, **5**, 195.

WASSERMAN, J., PETRINI, B. & BLOMGREN, H. (1982*b*). Radiosensitivity of T lymphocyte subpopulations. *J. Clin. Lab. Immunol.*, **7** 139.